# Spinal alignment measurement with Kinect sensor is valid for thoracic kyphosis but not for lumbar lordosis

**DOI:** 10.1186/s13018-023-03693-w

**Published:** 2023-03-19

**Authors:** Hitoshi Koda, Yoshihiro Kai, Noriyuki Kida, Toru Morihara

**Affiliations:** 1 Department of Rehabilitation Sciences, Faculty of Allied Health Sciences, Kansai University of Welfare Sciences, 3-11-1, Asahigaoka, Kashiwara-City, Osaka 582-0026 Japan; 2 Department of Physical Therapy, Faculty of Health Science, Kyoto Tachibana University, Kyoto, Japan; 3 Faculty of Arts and Sciences, Kyoto Institute of Technology University, Kyoto, Japan; 4 Department of Orthopedics, Marutamachi Rehabilitation Clinic, Kyoto, Japan

**Keywords:** Spinal alignment, Kinect sensor, Assessment

## Abstract

**Background:**

Spinal alignment evaluation is commonly performed in the clinical setting during rehabilitation. However, there is no simple method for its quantitative measurement. Recently, the depth cameras in Kinect sensors have been employed in various commercial and research projects in the healthcare field. We hypothesized that the time-of-flight technology of the Kinect sensor could be applied to quantitatively evaluate spinal alignment. The purpose of this study was to develop a simple and noninvasive evaluation for spinal alignment using the Kinect sensor and to investigate its validity.

**Methods:**

Twenty-four healthy men participated in the study. Measurement outcomes were the thoracic kyphosis and lumbar lordosis angles in the standing position, using a Spinal Mouse, the validity of which has been previously reported, and the Kinect sensor. In the measurement by the Kinect sensor, a program was created to obtain the three-dimensional coordinates of each point within an area marked on the monitor, and the sums of the angles at each vertebral level were calculated for the thoracic and lumbar areas. Pearson's correlation coefficient was used to analyze the relationship between the Kinect sensor and Spinal Mouse measurements of thoracic kyphosis and lumbar lordosis angles.

**Results:**

There was a significant positive and moderate correlation between the thoracic kyphosis measurements taken by each device. Contrarily, there was no significant correlation in the lordosis angle between measurements using the Kinect sensor and Spinal Mouse.

**Conclusions:**

Our results demonstrated the validity of measuring the thoracic kyphosis angle using the Kinect sensor. This indicates that the depth camera in the Kinect sensor is able to perform accurate thoracic alignment measurements quickly and noninvasively.

## Background

The trunk is located in the center of the body and plays an important role in stabilization, even when upper or lower limb movements are performed [1]. Abnormal spinal alignment is associated with pain and decreased mobility [2, 3]; it has been reported that poor spinal alignment was associated not only with spinal disorders but also with throwing shoulder disorders or osteoarthritis of the lower limbs [4, 5]. It is reported that adequate spinal alignment is needed to prevent or treat disorders [6].

In the clinical environment, the evaluation of spinal alignment has been traditionally performed using inspection and palpation by doctors or physical therapists. Plain radiographs or the Spinal Mouse have also been used to measure the angle of the spinal curve. However, the radiation exposure associated with radiographs and the high device costs limit their convenience. Therefore, there is still a need to establish simple and noninvasive assessment methods for clinical use.

We focused on the depth cameras in Kinect sensors, which are simple and inexpensive. Originally, the Kinect sensor was used as a game console operated without a controller. Recently, it has been employed in various commercial and research projects in the healthcare field [7, 8]. For example, it has been applied in medical settings as a gait analysis evaluation tool for patients with hemiplegia after stroke and to implement an exercise program for home use [9, 10]. The time-of-flight (ToF) technology installed in the Kinect sensor calculates distances based on the time difference between light emission and its reflection off of the subjects. Thus, the infrared Kinect sensor can reconstruct a three-dimensional image quickly and noninvasively. We hypothesized that quantitative spinal alignment evaluation could be possible by applying the ToF technology in the Kinect sensor.

The purpose of this study was to develop a simple and noninvasive spinal alignment evaluation using the Kinect sensor and to investigate its validity.

## Methods

Twenty-four healthy men participated in the study. Their mean age was 20.7 ± 0.5 years, mean height was 175.1 ± 6.9 cm, mean body weight was 65.8 ± 8.0 kg, and mean body mass index (BMI) was 21.4 ± 1.4 kg/m^2^. No subject had a history of spinal injuries or scoliosis before participating in this study. The study was performed in accordance with the World Medical Association’s Declaration of Helsinki. The purpose, nature, and potential risks of the experiments were fully explained to the participants, and all participants gave written informed consent prior to their inclusion in the study. This research has been approved by the Institutional Review Board of the authors’ affiliated institution.

The measurement outcomes were the thoracic kyphosis and lumbar lordosis angles. These were determined using a Spinal Mouse (Idiag, Voletswil Company, Switzerland), the validity of which has been previously reported [11], and the Kinect sensor (Microsoft, Redmond, WA, USA). For each measurement, the subjects were in the resting standing position and were instructed not to change their posture during the measurements. To avoid the influence of clothing, measurements were performed shirtless.

**Fig. 1:**
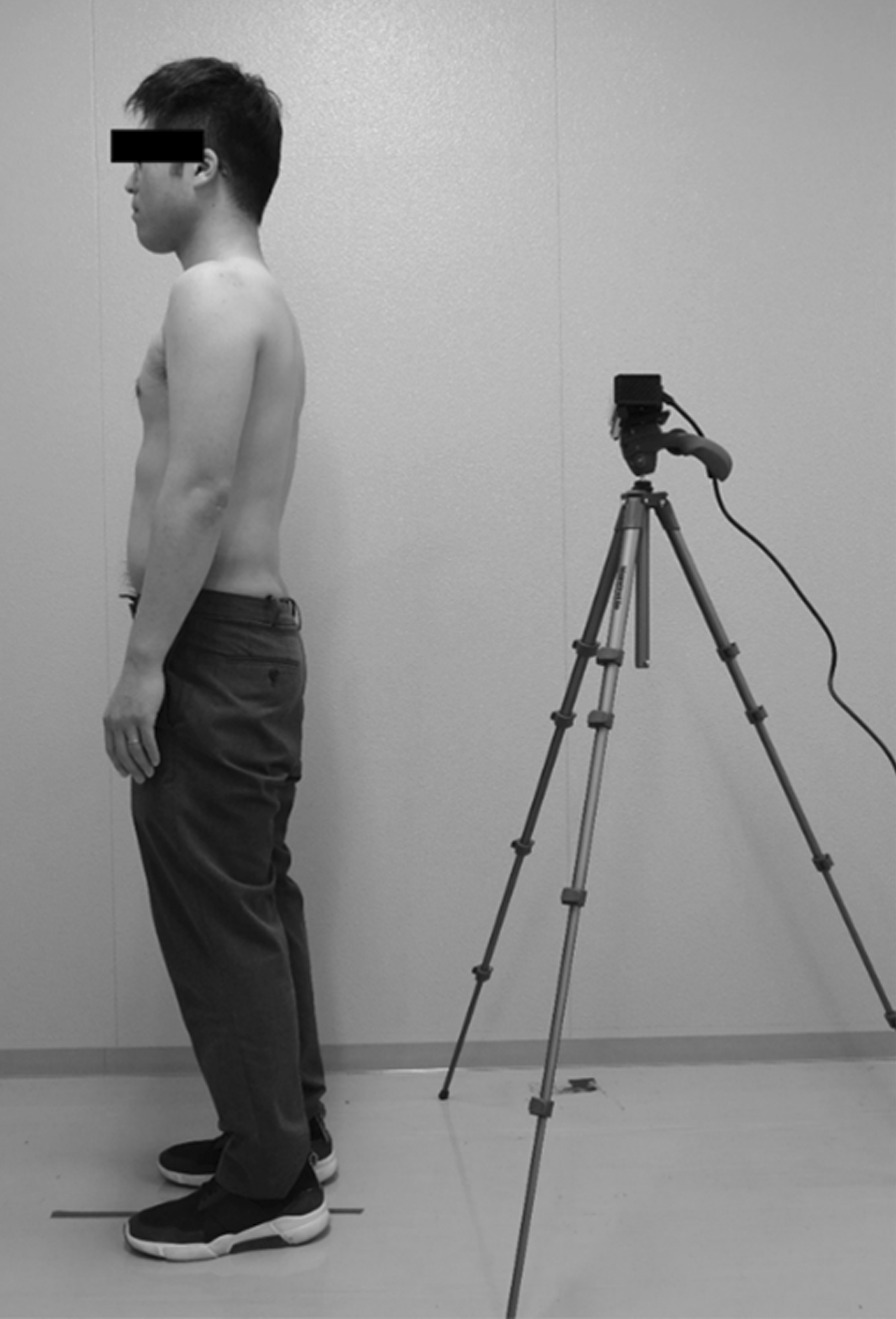
Measurements of three-dimensional data using the Kinect sensor

The three-dimensional data from the Kinect sensor were analyzed using Visual Studio (Microsoft, Redmond, WA, USA). The measuring areas were the first to twelfth thoracic vertebrae and the first to fifth lumbar vertebrae. The spinous processes of Th1, Th12, L1, and L5 were marked by prior palpation as anatomical landmarks. The Kinect sensor was placed 1 m behind the participants. Prior to measurement, two points in front of and behind the floor surface on the monitor were plotted as calibration points, and the vertical axis of the line connecting the two points was defined using the outer product of these segments. A program was created in Visual Studio to read the three-dimensional coordinates in the marked rectangular area on the screen. The Y (anteroposterior direction) and Z (vertical direction) coordinates were extracted from a row of plots located at the left–right center line in each of the thoracic and lumbar spine rectangular areas. The inner product of the two segments was used to calculate the angle between the upper and lower plots (Fig. 1). The direction (kyphosis or lordosis) was determined from the slope between the upper and lower plots. The thoracic kyphosis angles (Th1–12) and lumbar lordosis angles (L1–5) were determined by calculating the sum of the angles between each vertebral pair. Positive and negative signs were defined so that the kyphosis angle was positive in the thoracic spine and the lordosis angle was positive in the lumbar spine. In accordance with a previous study using the ToF method of the Kinect sensor, measurements were performed twice and the values of the second measurement were used for analysis [12].

Measurements of spinal alignment using the Spinal Mouse were taken after the Kinect measurements without changing the subject's position. The Spinal Mouse measures spinal curvature angles from the body surface, and its reliability and validity have already been confirmed [11]. The vertebral levels and positive direction of the angles were the same as for the Kinect sensor. Measurements were taken three times, and the average values obtained were used. Measurements were taken by a physical therapist with sufficient experience.

SPSS for Windows version 28.0 (IBM Corp., Armonk, NY, USA) was used for statistical analysis. Pearson's correlation coefficient was used to analyze the relationship between the Kinect sensor and Spinal Mouse measurements of thoracic kyphosis and lumbar lordosis angles. Statistical significance was set at *p* < 0.05.

## Results

**Fig. 2:**
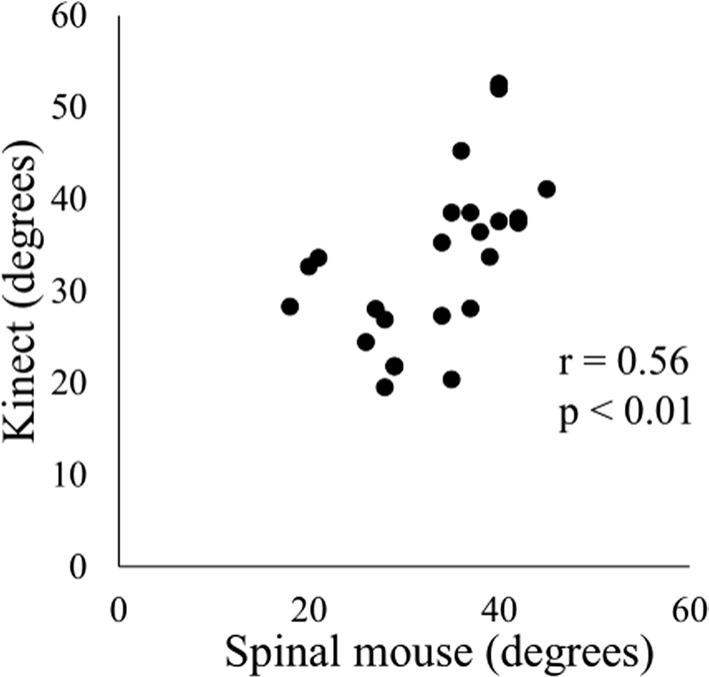
Correlation between the Kinect sensor and Spinal Mouse in measuring the thoracic kyphosis angle

The average thoracic kyphosis angles measured by the Kinect sensor and Spinal Mouse were 33.3 ± 9.1 and 33.3 ± 7.4 degrees, respectively. There was a significant positive moderate correlation between devices in the thoracic kyphosis angle measurements (*r* = 0.56, *p* < 0.05; Fig. 2).

**Fig. 3:**
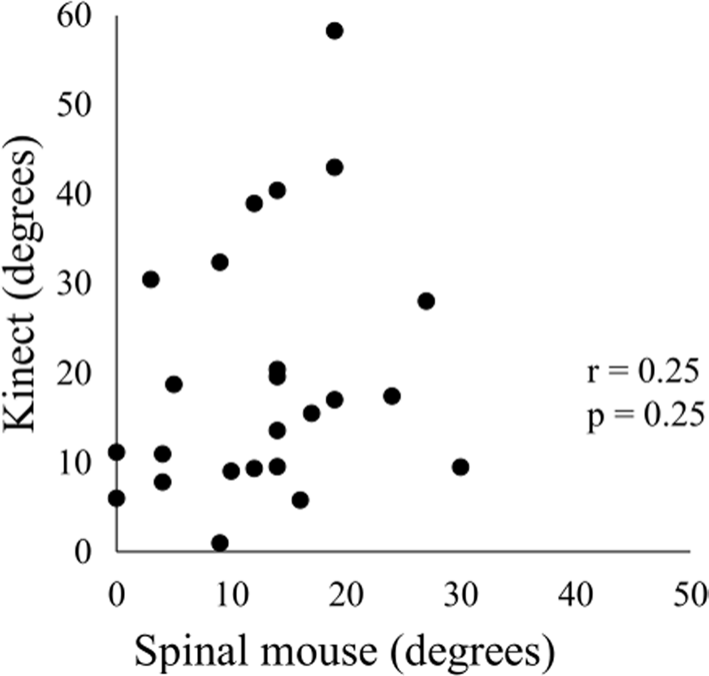
Correlation between the Kinect sensor and Spinal Mouse in measuring the lumbar lordosis angle

The lumbar lordosis angles measured by the Kinect sensor and Spinal Mouse were 19.7 ± 14.3 and 12.9 ± 7.9 degrees, respectively. There was no significant correlation between devices for the lumbar lordosis angle measurement (*r* = 0.25, *p* = 0.25; Fig. 3).

## Discussion

In this study, a noninvasive and simple method for evaluating trunk alignment was developed using Kinect. The results showed a significant moderate correlation between Kinect and Spinal Mouse for the measurement of the thoracic kyphosis angle. Conversely, the lumbar lordosis angle showed no significant correlation between the methods.

The thoracic kyphosis angle and mobility affect the movement of the scapula and upper limbs [13, 14]. A hyperkyphotic thoracic alignment has been suggested to play a role in rotator cuff tears, shoulder pain, little league shoulder, and limitation of upper limb elevation [15, 16]. It has been reported that shoulder pain and upper limb function improve after correction of excessive thoracic kyphosis [17]. Despite the importance of assessing the kyphosis angle, there is no simple method of its measurement in the clinical setting, and postural assessment by visual examination and palpation have been traditionally relied on. Our results demonstrated the validity of measuring the thoracic kyphosis angle using the Kinect sensor. Therefore, we suggest that it can be used as a simple and quantitative posture evaluation method.

On the contrary, there was no significant correlation in the lumbar lordosis angle measurement between Kinect sensor and Spinal Mouse. The lumbar lordosis alignment has been regarded as a factor in the development of low back pain or hip pain [18, 19]. The Spinal Mouse has been shown to be a reliable and valid tool for measuring the lumbar lordosis angles [20]. However, a poor association between Spinal Mouse and X-ray values in the lower thoracic and lumbar spine areas has been reported [21]. Moreover, three validated noninvasive instruments were shown to indicate different values in lumbar lordosis angle [22]. Further studies are warranted to elucidate these discrepancies. In the lumbar region, there is a wide range of thick tendons in the superficial layer, such as those of the erector spinae, longissimus dorsi, and iliacus muscles [23]. The shape of the superficial layer of the lumbar region read by the Kinect sensor might reflect the subcutaneous and muscle tissue contour around the lumbar spine. Therefore, it might be difficult to perform a quantitative evaluation of the lumbar area using the Kinect sensor.

The study’s limitations include the fact that the mean BMI of the study participants was 21.4 ± 1.4 (19.1–23.7) kg/m^2^. For subjects with a BMI > 24 kg/m^2^, this method derived from the surface contour might have limited applicability.

## Conclusions

The present study has thus shown that thoracic kyphosis alignment could be measured simply and quantitatively by using the Kinect sensor, but the same cannot be said for the lumbar lordosis alignment. This method can therefore be used as a simple and quantitative posture evaluation method in the thoracic spine.

## Data Availability

The datasets used and/or analyzed during the current study are available from the corresponding author on reasonable request.

## Abbreviations

BMI: Body mass index
ToF: Time-of-flight
